# Early Outcomes and Mid-Term Follow-Up of Melody TPV Implantation: A Ten-Year Single-Center Retrospective Observational Study

**DOI:** 10.3390/healthcare14121699

**Published:** 2026-06-15

**Authors:** Mario Giordano, Gianpiero Gaio, Raffaella Marzullo, Ippolita Altobelli, Raffaele Barbato, Raffaella Esposito, Giancarlo Scognamiglio, Gabriella Gaudieri, Michela Palma, Maurizio Cappelli Bigazzi, Giuseppe Limongelli, Berardo Sarubbi, Maria Giovanna Russo

**Affiliations:** 1 Paediatric Cardiology Unit, Monaldi Hospital, “A.O. dei Colli”, University of Campania “Luigi Vanvitelli”, 80138 Naples, Italy; 2 Adult Congenital Heart Disease Unit, Monaldi Hospital, “A.O. dei Colli”, University of Campania “Luigi Vanvitelli”, 80138 Naples, Italy; 3 Invasive Cardiology Unit, Monaldi Hospital, “A.O. dei Colli”, University of Campania “Luigi Vanvitelli”, 80138 Naples, Italy; 4 Department of Translational Medical Sciences, Inherited and Rare Cardiovascular Diseases, Monaldi Hospital, “A.O. dei Colli”, University of Campania “Luigi Vanvitelli”, 80138 Naples, Italy

**Keywords:** Melody TPV, pulmonary valve replacement, cardiac catheterization, congenital heart diseases

## Abstract

**Introduction**: The Melody transcatheter pulmonary valve (TPV) was the first percutaneous bioprosthetic valve approved for transcatheter pulmonary valve implantation (TPVI). We report our single-centre experience with Melody TPV implantation in patients with congenital heart disease (CHD). **Methods**: This retrospective observational single-centre study included all patients evaluated in the catheterization laboratory for Melody TPV implantation. Early outcomes included procedural failure, life-threatening adverse events, and mortality. Long-term outcomes assessed during follow-up included infective endocarditis, transcatheter reintervention, and surgical reintervention. **Results**: Between 2015 and 2025, 50 consecutive patients were evaluated for TPVI with the Melody TPV at our institution. In four patients (8%), the procedure was aborted because of coronary artery compression detected during balloon interrogation of the right ventricular outflow tract (RVOT). One patient (2%) died of septic shock following acute pulmonary oedema in the immediate post-procedural period. The remaining 45 patients (90%) underwent successful Melody TPV implantation and were discharged from hospital. In six patients, the Melody TPV was implanted off-label: in the tricuspid position (n = 2) and in small conduits (<16 mm) (n = 4). Mean follow-up duration was 5.8 ± 3.6 years. One patient was lost to follow-up. Among the remaining 44 patients, seven (15.9%; 2.7% per patient-year) developed infective endocarditis, seven (15.9%; 2.7% per patient-year) underwent transcatheter reintervention (six balloon dilatations of the Melody valve and one valve-in-valve implantation), and four (9.1%; 1.5% per patient-year) required surgical replacement of the Melody TPV. **Conclusions**: Transcatheter implantation of the Melody TPV is an effective treatment for RVOT dysfunction. At mid-term follow-up, the majority of implanted Melody valves demonstrated satisfactory function, and only a minority of patients required surgical valve replacement.

## 1. Introduction

The Melody Transcatheter Pulmonary Valve (TPV) (Medtronic Inc., Minneapolis, MN, USA) was the first percutaneous bioprosthetic valve specifically developed for implantation in the pulmonary position [1]. It is a balloon-expandable biological valve composed of a bovine jugular vein containing a native venous valve, sutured within a closed-cell Cheatham-Platinum (CP) stent (NuMED Inc., Hopkinton, NY, USA) [2].

According to the manufacturer’s instructions for use (IFU), the Melody TPV is indicated for the treatment of dysfunctional right ventricular outflow tract (RVOT) conduits or failed bioprosthetic valves with diameters ranging from 16 to 22 mm. Nevertheless, several studies have reported its off-label use in small conduits (diameter < 16 mm) [3,4] and in the native RVOT [5]. As a result, implantation strategies have evolved over time.

During the early experience with the Melody TPV, direct implantation without pre-stenting was commonly performed. In contemporary practice, however, routine pre-stenting is generally recommended to reduce the risk of stent fracture and enhance long-term valve durability [6]. Direct implantation is currently reserved primarily for valve-in-valve procedures or for non-distensible conduits supported by an external rigid frame (e.g., the Hancock conduit; Medtronic Inc., Minneapolis, MN, USA) [7].

Historically, surgical repair was the only available treatment option for relieving right ventricular outflow tract (RVOT) dysfunction. Nowadays, transcatheter pulmonary valve implantation (TPVI) has become the preferred therapeutic strategy in this setting [8,9]. The Melody TPV remains the only transcatheter pulmonary bioprosthesis specifically designed for implantation in patients with a small pulmonary annulus (<22 mm) [2].

The aim of this study was to assess early outcomes and mid-term results following Melody TPV implantation, with particular emphasis on procedural failure, procedure-related complications, and the incidence of infective endocarditis, as well as transcatheter and surgical reinterventions during follow-up.

## 2. Materials and Methods

### 2.1. Study Design and Population

This retrospective, observational, single-centre study was conducted over a 10-year period.

At our institution (Paediatric Cardiology Unit and Adult Congenital Heart Disease Unit, Monaldi Hospital, “Ospedali dei Colli”, Naples, Italy), all patients with repaired congenital heart disease (CHD) presenting with significant residual RVOT obstruction and/or pulmonary valve regurgitation underwent multidisciplinary Heart Team evaluation to determine the most appropriate treatment strategy, including surgical or transcatheter intervention. Transcatheter treatment was preferentially considered when deemed technically feasible based on pre-procedural imaging assessment. Indications for intervention were established in accordance with the latest European Society of Cardiology (ESC) Guidelines for the Management of Adult Congenital Heart Disease [9]. Between 2015 and 2025, all consecutive patients considered suitable candidates for Melody TPV implantation by the Heart Team underwent cardiac catheterization for TPV implantation at our institution and were included in the present study. Patients judged unsuitable for Melody TPV implantation and referred for alternative treatment strategies were excluded from the analysis.

Demographic and clinical data were retrospectively collected from medical records.

Patients were stratified into six major CHD categories: tetralogy of Fallot (ToF) (Group I); pulmonary atresia with ventricular septal defect (PA-VSD) and truncus arteriosus (TA) (Group II); pulmonary stenosis (PS) (Group III); previous Ross procedure (Group IV); transposition of the great arteries (TGA), including both dextro-transposition of the great arteries (d-TGA) and congenitally corrected transposition of the great arteries (ccTGA) (Group V); and other CHDs (Group VI).

The type, size, and anatomical location of conduits and/or bioprosthetic valves were recorded. The mechanism of conduit or bioprosthetic valve dysfunction was also assessed and classified as predominant stenosis, predominant regurgitation, or mixed dysfunction.

Early outcomes assessed during hospitalization for Melody TPV implantation included procedural failure, procedure-related life-threatening adverse events, and mortality. Procedural failure was defined as any unsuccessful attempt to implant the Melody TPV. Procedure-related life-threatening adverse events were defined as complications occurring during or after the procedure that had the potential to result in patient death. Minor adverse events were also recorded.

Patients who underwent Melody TPV implantation were scheduled for regular follow-up, including clinical evaluation, electrocardiography (ECG), and transthoracic echocardiography. The first follow-up evaluation was one-month after the procedure, whereas the other ones six-months from the last visit. Patients lost to follow-up were excluded from the long-term outcome analysis. Long-term outcomes assessed at follow-up included infective endocarditis and the need for reintervention, either transcatheter or surgical. Infective endocarditis was diagnosed according to the most recent ESC guidelines [10].

Potential risk factors for infective endocarditis and reintervention were analyzed, including sex, age and body weight at the time of Melody TPV implantation, CHD category, conduit type and size, and conduit anatomical location (anatomical versus extra-anatomical).

### 2.2. Patient and Public Involvement

Given the retrospective nature of the study, no patient or public involvement was deemed necessary. Accordingly, patients and the public were not involved in the design, conduct, reporting, or dissemination of this research.

### 2.3. Statistical Analysis

All statistical analyses were performed using the Statistical Package for the Social Sciences (SPSS) for Windows, version 20 (IBM Corp., Chicago, IL, USA). Continuous variables (e.g., weight and age) were expressed as mean ± standard deviation or as median with interquartile range (IQR), as appropriate. Categorical variables were presented as percentages. Univariate analysis of potential risk factors was performed using Student’s *t*-test for continuous variables and the chi-square test for categorical variables. Kaplan–Meier curves were used to assess the freedom from the long-term outcomes over time and to estimate event rates per year. A *p*-value ≤ 0.05 was considered statistically significant.

### 2.4. Melody TPV Implantation Procedure

The interventional procedure is performed via venous vascular access, usually through the femoral vein. At the beginning of the procedure, sodium heparin (100 IU/kg, up to a maximum dose of 5000 IU) is administered. The anatomy of the dysfunctional RVOT is assessed by right ventricular and pulmonary angiography. Aortic and/or selective coronary angiography is performed to evaluate coronary artery anatomy and its spatial relationship to the RVOT and pulmonary artery. Balloon interrogation of the RVOT is performed to predict expansion of the landing zone and to minimize the risk of coronary artery and/or aortic root compression. The balloon selected for this test should be capable of dilating the RVOT conduit to the intended final diameter of the Melody TPV, thereby allowing accurate assessment of its relationship with the coronary arteries. Following a negative balloon interrogation test, a CP stent is typically implanted to dilate the dysfunctional RVOT conduit and optimize the landing zone. Balloon interrogation and pre-stenting are generally not performed in valve-in-valve procedures or in non-distensible conduits with an external rigid support (e.g., Hancock conduits). After pre-stenting, the Ensemble II delivery system (Medtronic Inc., Minneapolis, MN, USA) is advanced across the landing zone, and the Melody TPV is deployed. Following valve implantation, intravenous acetylsalicylic acid (300 mg) is administered.

## 3. Results

### 3.1. Demographic, Procedural, and Clinical Features and Early Outcomes

Between 2015 and 2025, 50 patients with RVOT dysfunction underwent cardiac catheterization for evaluation of Melody TPV implantation. Mean body weight and age were 61.6 ± 21.3 kg (range 23–130 kg) and 22.1 ± 12.0 years (range 8–66 years), respectively. The majority of patients had tetralogy of Fallot (ToF; Group I) (n = 34, 68%), followed by pulmonary atresia with ventricular septal defect or truncus arteriosus (PA-VSD/TA; Group II) (n = 5, 10%), pulmonary stenosis (PS; Group III) (n = 2, 4%), previous Ross procedure (Group IV) (n = 4, 8%), transposition of the great arteries (TGA; Group V) (n = 3, 6%), and other congenital heart diseases (Group VI) (n = 2, 4%).

In four patients (8%), Melody TPV implantation was not performed because of significant coronary artery compression detected during balloon interrogation of the RVOT. These patients were subsequently referred for elective surgical pulmonary valve replacement. In the remaining 46 patients (92%), Melody TPV implantation was successfully completed.

Among the patients who underwent Melody TPV implantation (n = 46), RVOT pre-stenting was performed in 33 cases (71.7%). Direct Melody TPV implantation was performed in 12 patients (26.1%) with a dysfunctional pulmonary bioprosthetic valve and in one patient (2.2%) with a Hancock conduit. The mean diameter of the dysfunctional conduit or bioprosthetic valve was 19.6 ± 3.1 mm (range 12–25 mm).

**Figure 1:**
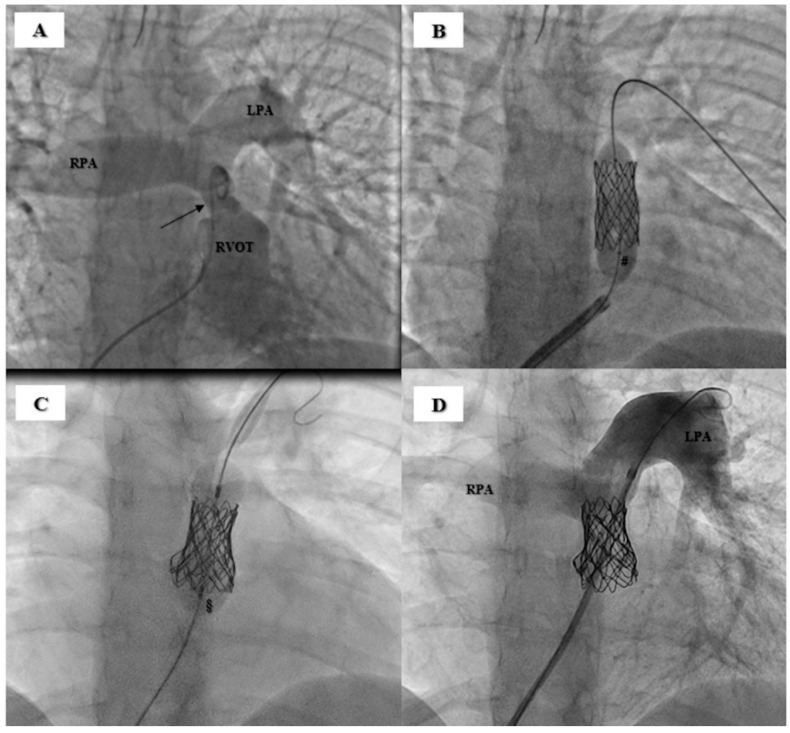
Off-label Melody TPV implantation to treat a dysfunctional small conduit. The right ventricle angiography highlighted a degenerated and stenotic small conduit (12 mm) (arrow) in pulmonary position (**A**). A pre-stenting by a covered CP stent (**#**) allows to dilatate the conduit and to optimize the landing zone (**B**). Melody TPV crimped onto its Ensemble II delivery system (**§**) is effectively implanted (**C**). After Melody TPV implantation, the pulmonary angiography highlights a continent bioprosthesis without pulmonary artery injuries (**D**). Abbreviations. LPA: Left Pulmonary Artery; RPA: Right Pulmonary Artery; RVOT: Right Ventricular Outflow Tract.

**Figure 2:**
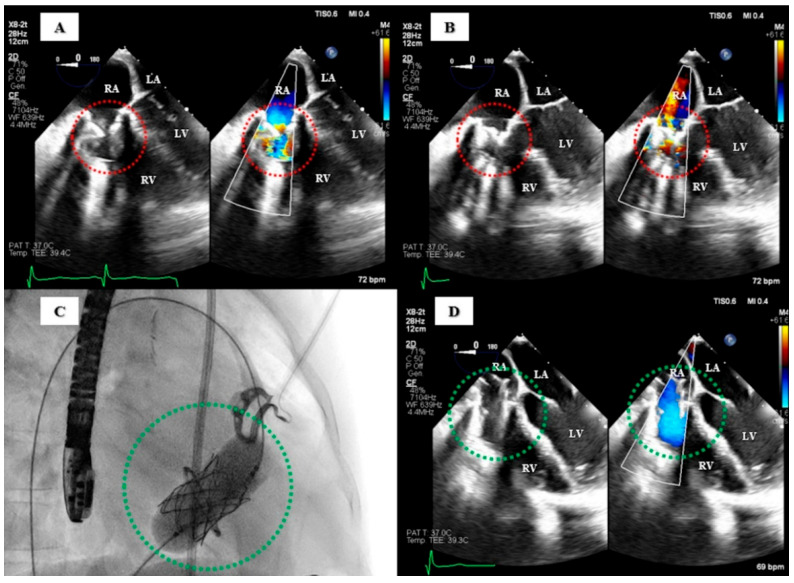
Off-label Melody TPV implantation in tricuspid position. The intra-procedural trans-esophageal echocardiography (0° mid-esophageal view) detects a degenerative biological valvular prosthesis (red circle) in tricuspid position with both a significant stenosis (**A**) as well as a regurgitation (**B**). The right lateral view angiography of Melody TPV (green circle) implantation inside the degenerated tricuspid bioprosthesis (**C**). After Melody TPV implantation, the intra-procedural trans-esophageal echocardiography (0° mid-esophageal view) highlights a good functioning Melody TPV (green circle) in tricuspid position without stenosis or regurgitation (**D**). Abbreviations. LA: Left Atrium; LV: Left Ventricle; RA: Right Atrium; RV: Right Ventricle.

The Melody TPV was implanted off-label in six patients. Specifically, four patients (8.7%) had small conduits (three 14-mm conduits and one 12-mm conduit), while two patients (4.3%) underwent implantation in the tricuspid position for dysfunctional bioprosthetic valves (Figure 1 and Figure 2). Both patients who received a Melody TPV in the tricuspid position belonged to CHD Group VI.

**Figure 3:**
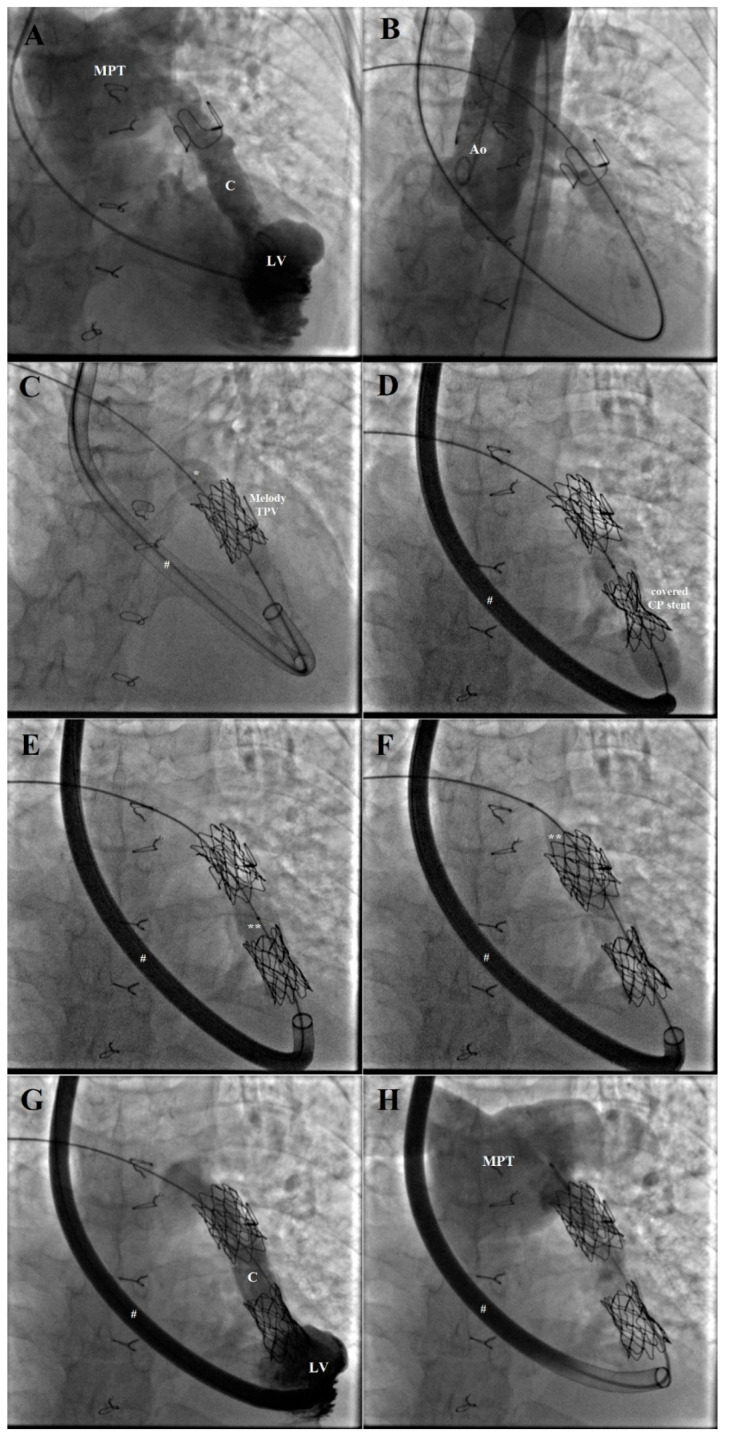
Extra-anatomical dysfunctional conduit treatment by percutaneous Melody TPV implantation via internal jugular vein. The sub-pulmonary ventricle angiography highlighted a calcific degeneration of the pulmonary conduit in extra-anatomic position (**A**). Balloon interrogation of the conduit demonstrated the absence of coronary compression or aortic root deformation (**B**). Melody TPV crimped onto a semi-compliant balloon (*) is effectively implanted using a hydrophilic Dryseal long delivery sheath (#) (**C**). A covered CP stent is released to deal the stenosis at the proximal anastomosis (**D**). A high-pressure balloon catheter (**) is used to post-dilate both the Melody TPV and the covered CP stent (**E**,**F**). Post-implantation right ventricle and pulmonary angiography highlighted a good angiographic result of the procedure with a residual mild pulmonary regurgitation (**G**,**H**). Abbreviations. Ao: Aorta. C: Conduit. LV: Left Ventricle. MPT: Main Pulmonary Trunk.

The femoral vein was the most commonly used vascular access site. However, the internal jugular vein was used in three patients (6.5%). Alternative venous access was required in one patient because of bilateral femoral vein thrombosis, whereas in the remaining two patients, the jugular approach was preferred because it provided a more favorable route for intervention in the presence of an extra-anatomical RVOT conduit (Figure 3).

Two procedure-related minor adverse events (4.3%) were observed: one femoral artery pseudoaneurysm requiring surgical repair and one small femoral arteriovenous fistula that resolved spontaneously following 24 h of compression bandaging.

One patient (2.2%) developed hyperacute pulmonary oedema shortly after successful Melody TPV implantation. Despite intensive medical treatment, the patient died 12 days later from septic shock.

The main demographic, clinical, and procedural data are summarized in Table 1.

### 3.2. Mid-Term Follow-Up

Among the 50 patients evaluated in the catheterization laboratory, 45 (90%) underwent successful Melody TPV implantation and were discharged from hospital. One patient was lost to follow-up. The remaining 44 patients had a mean follow-up duration of 5.8 ± 3.6 years.

During follow-up, seven patients (15.9%; 2.7% per patient-year) developed infective endocarditis at a mean of 4.41 ± 3.93 years after Melody TPV implantation. The causative microorganisms were *Staphylococcus epidermidis* (n = 3), *Staphylococcus aureus* (n = 2), *Enterococcus faecium* (n = 1), and *Brucella* spp. (n = 1). All patients received targeted antimicrobial therapy based on blood culture results, in accordance with the most recent ESC guidelines [8]. In four cases (57.1%), medical therapy resulted in complete resolution of infective endocarditis without recurrence during follow-up. In the remaining three patients (42.9%), surgical replacement of the Melody TPV was required. These cases of surgical replacement were associated with infective endocarditis caused by *S. epidermidis*, *E. faecium*, and *Brucella* spp., respectively.

**Figure 4:**
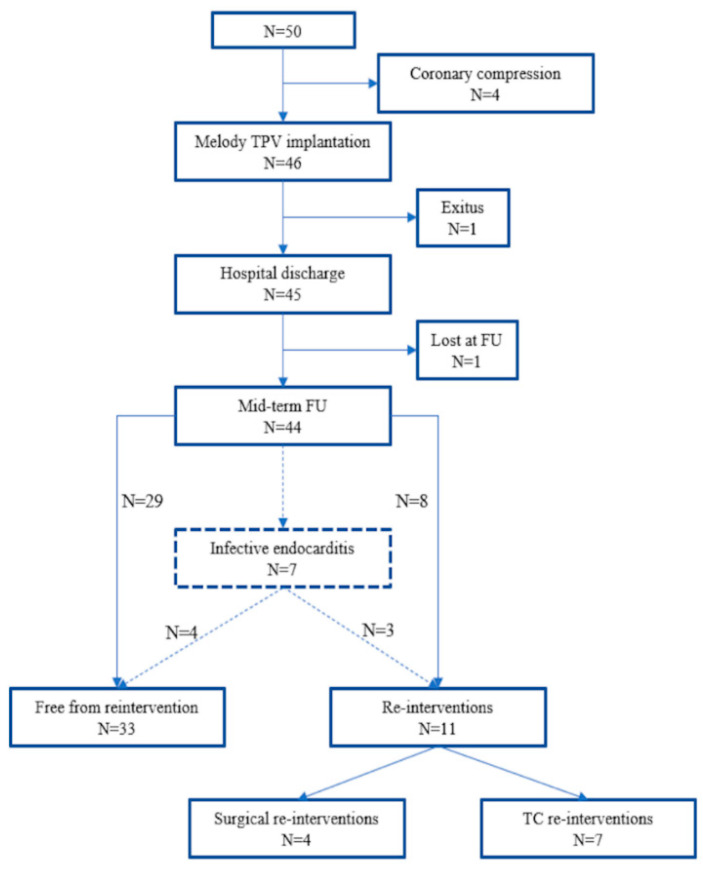
Flowchart of the study cohort. Abbreviations. FU: Follow-Up. TC: Trans-Catheter.

**Figure 5:**
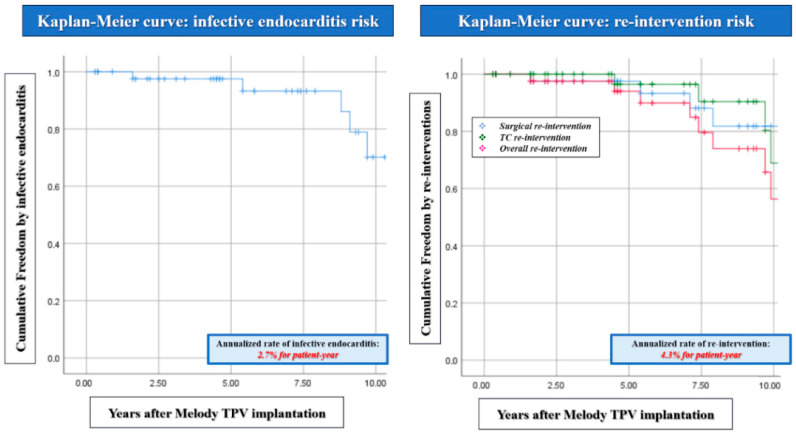
Kaplan–Meier curves about the risk of the first episode of infective endocarditis (**left box**) and the trans-catheter (TC), surgical and overall risk of re-interventions (**right box**) during follow-up.

Overall, 11 patients (25%; 4.3% per patient-year) required reintervention. Of these, seven patients (15.9%; 2.7% per patient-year) underwent transcatheter reintervention (six balloon dilations of the Melody valve and one valve-in-valve implantation), while four patients (9.1%; 1.5% per patient-year) underwent surgical replacement of the Melody TPV (three due to antibiotic-resistant infective endocarditis and one due to patient–prosthesis mismatch) (Figure 4 and Figure 5).

### 3.3. Risk Factor Analysis

At univariate analysis, sex (71.4% vs. 55.2% male, *p* = 0.4), body weight (62.4 ± 13.3 vs. 84.5 ± 16.3 kg, *p* = 0.9), age at the time of the procedure (21.6 ± 6.0 vs. 25.2 ± 9.1 years, *p* = 0.8), CHD group (*p* = 0.2), and conduit/bioprosthesis diameter (20.6 ± 2.6 vs. 19.7 ± 1.6 mm, *p* = 0.3) were not significantly associated with an increased risk of infective endocarditis. Furthermore, no significant differences in the risk of infective endocarditis were observed between conduits and bioprosthetic valves (17.9% vs. 20%, *p* = 0.9) or among different conduit types (*p* = 0.4).

Similarly, at univariate analysis, sex (54.5% vs. 54% male, *p* = 0.99), body weight (56.0 ± 22.0 vs. 64.6 ± 21.8 kg, *p* = 0.3), age at the time of the procedure (19.6 ± 11.2 vs. 22.5 ± 12.3 years, *p* = 0.5), CHD group (*p* = 0.7), and conduit/bioprosthesis diameter (18.8 ± 2.2 vs. 20.2 ± 3.2 mm, *p* = 0.1) were not significantly associated with an increased risk of transcatheter or surgical reintervention. Moreover, no significant differences in reintervention rates were observed between conduits and bioprosthetic valves (25.8% vs. 21.4%, *p* = 0.8) or among different conduit types (*p* = 0.5).

## 4. Discussion

The Melody TPV was the first percutaneous valvular bioprosthesis specifically designed for implantation in the pulmonary position [1]. Initially, its use was limited to dysfunctional RVOT conduits or bioprosthetic valves; however, its clinical application has progressively expanded over time. Several studies have demonstrated the safety and efficacy of Melody TPV implantation beyond the IFU, including off-label applications in small conduits (diameter < 16 mm) [3,4] and in the native RVOT [5].

Although the Melody transcatheter pulmonary valve TPV provides acceptable hemodynamic performance at diameters ≥ 16 mm, the expandable nature of compliant bovine jugular vein conduits, such as the Contegra conduit (Medtronic, Inc., Minneapolis, MN, USA), permits dilation to larger diameters, thereby optimizing valve function and durability [3]. In these cases, pre-stenting is considered mandatory, not only to establish an optimal landing zone for valve implantation but also because the radial force exerted by the stent enables durable conduit remodeling and maintenance of the achieved diameter over time. High-pressure non-compliant balloon inflation is often required to adequately expand the conduit, the stent scaffold, and/or the Melody TPV.

The large size of dysfunctional native RVOTs frequently limits the applicability of the Melody TPV, often favoring the use of alternative transcatheter pulmonary valve prostheses specifically designed for larger outflow tract anatomies [2]. Expansion of the Melody TPV beyond 24 mm is associated with a substantial risk of valve incompetence, potentially requiring repeat intervention. However, in carefully selected patients with native RVOT diameters < 24 mm, Melody TPV implantation has been shown to provide effective valve function and durable long-term clinical outcomes [5,11].

More recently, its use has also been extended to the treatment of dysfunctional bioprosthetic valves in the tricuspid position [12]. However, few data are available about the long-term follow-up of the Melody TPV implantation in this setting.

Our findings suggest the effectiveness of Melody TPV implantation both within and beyond the IFU; however, further evidence is required to confirm the long-term outcomes of off-label Melody TPV implantation. The most common cause of failed implantation in our series was coronary artery compression detected during balloon interrogation. Notably, patients with extra-anatomical conduits between the RVOT and pulmonary artery exhibited a significantly higher risk of coronary compression. Therefore, RVOT balloon interrogation remains a critical step to prevent this life-threatening complication. In high-risk patients, advanced imaging techniques, such as holographic simulation, have emerged as promising tools to pre-procedurally assess conduit deformation and calcification displacement during valve deployment [13]. Similarly, three-dimensional printed models may provide valuable insights into RVOT anatomy and its spatial relationship with the coronary arteries [14].

In our cohort, one major adverse event was observed: a case of hyperacute pulmonary oedema. This complication is particularly relevant in patients with elevated pulmonary capillary wedge pressure and severe pulmonary regurgitation. Successful Melody TPV implantation increases effective forward pulmonary blood flow by eliminating regurgitant flow. In patients with elevated filling pressures, this abrupt increase in antegrade pulmonary flow may precipitate acute pulmonary oedema. In such cases, pre-procedural optimization with diuretic therapy may help mitigate this risk [15].

The annualized incidence of Melody TPV-related infective endocarditis in our series was 2.7% per patient-year, highlighting infective endocarditis as a persistent clinical concern. However, only 43% of affected patients required surgical valve replacement, while in the majority of cases, targeted antimicrobial therapy alone was sufficient to control the infection. Recent studies suggest that prolonged acetylsalicylic acid therapy, implantation at age > 12 years, and a residual peak-to-peak gradient < 15 mmHg are associated with a lower risk of infective endocarditis [16,17,18,19]. Future improvements in implantation techniques, long-term antiplatelet therapy, and stricter attention to dental hygiene may further reduce this risk [20].

At mid-term follow-up, the majority of patients remained free from reintervention. In most cases, transcatheter approaches were sufficient to manage Melody TPV dysfunction. Surgical reintervention was required in a minority of patients, most commonly due to antimicrobial-resistant infective endocarditis. Improved management of infective endocarditis, along with enhanced preventive strategies, may further reduce the need for surgical valve replacement [20,21].

The most common transcatheter reintervention in our series was balloon dilation. Stent recoil represents a major mechanism of Melody TPV restenosis, and the use of high-pressure non-compliant balloons can effectively restore an adequate valve diameter and function. In selected cases, leaflet degeneration may necessitate valve-in-valve implantation. In earlier experiences, stent fractures were a frequent cause of valve dysfunction; however, in the current era of systematic pre-stenting, the incidence of Melody TPV fracture has markedly decreased [21,22,23,24,25].

## 5. Study Limitations

This is a retrospective, observational, single-centre study, which inherently limits the generalisability of the findings. Although clinically relevant, the results should be interpreted with caution, as the small sample size may limit the robustness of the univariate analysis of risk factors. Larger, multicentre international studies are needed to confirm these findings and provide more robust evidence.

Furthermore, the development of prospective registries may enable more comprehensive and systematic analyses in this relatively small and heterogeneous patient population. Finally, over the past decade, procedural techniques have likely evolved and improved, potentially influencing procedural outcomes. A multicentre study design would allow for a larger cohort and enable stratified analyses of outcomes according to implantation period.

## 6. Conclusions

The Melody TPV is an effective percutaneous bioprosthetic valve with a high procedural success rate. The femoral vein represents the preferred vascular access; however, alternative venous approaches, such as the internal jugular vein, may be used when clinically indicated.

At mid-term follow-up, a high proportion of patients remain free from Melody-related adverse events, mortality, and surgical reintervention. Transcatheter reinterventions are most commonly due to stent recoil and can usually be managed with percutaneous balloon dilation. In cases of leaflet degeneration, a valve-in-valve procedure represents an effective therapeutic option.

## Figures and Tables

**Table 1:** Demographic, clinical and procedural data.

**Demographical and Clinical Features**	
**Overall Cohort**	**N = 50**
Weight (kg)	61.6 ± 21.3
Age (years)	22.1 ± 12
CHD group:	
- group I	34 (68%)
- group II	5 (10%)
- group III	2 (4%)
- group IV	4 (8%)
- group V	3 (6%)
- group VI	2 (4%)
Dysfunctional RVOT mechanism	
- stenosis	39 (78%)
- regurgitation	4 (8%)
- stenosis and regurgitation	5 (10%)
Tricuspid stenosis	2 (4%)
RVOT-PA conduit	31 (62%)
Pulmonary valve bioprosthesis	17 (34%)
Tricuspid valve bioprosthesis	2 (4%)
Conduit/bioprosthesis size (mm)	19.6 ± 3.1
Extra-anatomical VD-PA conduit	6 (12%)
Coronary compression (BIT)	4 (8%)
**Successful Melody TPV implantation**	**N = 46**
Vascular access	
- femoral vein	43 (93.5%)
- internal jugular vein	3 (6.5%)
Pre-stenting	33 (71.7%)
No pre-stenting	13 (28.3%)
Off label Melody TPV implantation	6 (13%)
- small conduits	4 (8.7%)
- TV implantation	2 (4.3%)
Adverse events	
- femoral pseudoaneurysm	2 (4.3%)
- hyperacute pulmonary edema	1 (2.2%)

Abbreviations. BIT: balloon interrogation test; CHD: congenital heart disease; PA: pulmonary artery; RVOT: right ventricle outflow tract; TV: tricuspid valve.

## Data Availability

The original contributions presented in this study are included in the article. Further inquiries can be directed to the corresponding author.
